# Highly Efficient Antimicrobial Activity of Cu*_x_*Fe*_y_*O*_z_* Nanoparticles against Important Human Pathogens

**DOI:** 10.3390/nano10112294

**Published:** 2020-11-20

**Authors:** Lu Zhu, David W. Pearson, Stéphane L. Benoit, Jing Xie, Jitendra Pant, Yanjun Yang, Arnab Mondal, Hitesh Handa, Jane Y. Howe, Yen-Con Hung, Jorge E. Vidal, Robert J. Maier, Yiping Zhao

**Affiliations:** 1 School of Chemical, Materials and Biomedical Engineering, College of Engineering, University of Georgia, Athens, GA 30602, USA; zhulu5466@gmail.com (L.Z.); jp50261@uga.edu (J.P.); YanjunYang@uga.edu (Y.Y.); Arnab.Mondal@uga.edu (A.M.); hhanda@uga.edu (H.H.); 2 Department of Physics and Astronomy, University of Georgia, Athens, GA 30602, USA; davidwpuga@gmail.com; 3 Department of Microbiology, University of Georgia, Athens, GA 30602, USA; stefbens@uga.edu (S.L.B.); rmaier@uga.edu (R.J.M.); 4 Department of Food Science & Technology, University of Georgia, Griffin, GA 30223, USA; jing.xie25@uga.edu (J.X.); yhung@uga.edu (Y.-C.H.); 5 Department of Materials Science & Engineering, University of Toronto, Toronto, ON M5S 1A1, Canada; jane.howe@utoronto.ca; 6 Department of Microbiology and Immunology, University of Mississippi, Jackson, MS 39216, USA; jvidal@umc.edu

**Keywords:** antimicrobial materials, inorganic nanomaterials, drug-resistant bacteria, metal oxides, copper iron oxides, copper oxide, cuprous oxide

## Abstract

The development of innovative antimicrobial materials is crucial in thwarting infectious diseases caused by microbes, as drug-resistant pathogens are increasing in both number and capacity to detoxify the antimicrobial drugs used today. An ideal antimicrobial material should inhibit a wide variety of bacteria in a short period of time, be less or not toxic to normal cells, and the fabrication or synthesis process should be cheap and easy. We report a one-step microwave-assisted hydrothermal synthesis of mixed composite Cu*_x_*Fe*_y_*O*_z_* (Fe_2_O_3_/Cu_2_O/CuO/CuFe_2_O) nanoparticles (NPs) as an excellent antimicrobial material. The 1 mg/mL Cu*_x_*Fe*_y_*O*_z_* NPs with the composition 36% CuFeO_2_, 28% Cu_2_O and 36% Fe_2_O_3_ have a general antimicrobial activity greater than 5 log reduction within 4 h against nine important human pathogenic bacteria (including drug-resistant bacteria as well as Gram-positive and Gram-negative strains). For example, they induced a >9 log reduction in *Escherichia coli* B viability after 15 min of incubation, and an ~8 log reduction in multidrug-resistant *Klebsiella pneumoniae* after 4 h incubation. Cytotoxicity tests against mouse fibroblast cells showed about 74% viability when exposed to 1 mg/mL Cu*_x_*Fe*_y_*O*_z_* NPs for 24 h, compared to the 20% viability for 1 mg/mL pure Cu_2_O NPs synthesized by the same method. These results show that the Cu*_x_*Fe*_y_*O*_z_* composite NPs are a highly efficient, low-toxicity and cheap antimicrobial material that has promising potential for applications in medical and food safety.

## 1. Introduction

Infectious diseases are the second leading cause of death in the world (third in the United States) and the leading cause of disability-adjusted life years worldwide (1 disability-adjusted life year is one lost year of healthy life) [1,2,3,4,5]. Bacterial-mediated infections, including those due to *Salmonella*, *Escherichia coli*, or *Shigella*, contribute to a large portion of overall infections, including, for example, the recent outbreak of *Listeria* infections linked to Deli ham [6] and *Salmonella* infections linked to ground beef [7]. Penicillin, and subsequently other antibiotics, have been critical for combating pathogens, but the increased antibiotic resistance observed in these pathogens has become an extremely serious public health concern [8]. Even when drug resistance is not the factor limiting their use, other disadvantages of antibiotics, such as their toxicity to the host, lead to the additional need to explore the efficacy of novel antimicrobial reagents [9]. New antimicrobial approaches other than the use of antibiotics have been proposed and developed, some of which include photodynamic therapy [10] and bacteria inactivation by nanoparticles [11,12]. In particular, with the advance of nanotechnology, the use of nano-sized inorganic materials as antimicrobial agents has attracted increasing attention for the control of the microbes [13,14,15,16,17]. The inorganic antimicrobial materials can be divided into two major categories based on their action mechanisms—the photocatalytic antimicrobial materials (activated by light), and nanomaterials that can directly lead to bacteria elimination or death (direct antimicrobial nanomaterials). Due to the advanced oxidation processes induced by photocatalytic properties, metal oxide-based photocatalytic nanomaterials have shown great promise as effective non-targeted disinfectants against a wide range of microorganisms and the decomposition of chemical contaminants [18,19,20,21,22,23,24,25,26,27,28]. The requirement of different wavelengths of light to activate the bactericidal activity of these materials has pros and cons; on one hand, light can be used to control the activity of the materials, and on the other hand, the nature of the materials limits the use of the light, the system requires additional accessories to generate light, and the material is not effective for applications in dark environments. There are also metal/metal oxide/2D nanostructures which can produce ions toxic to bacteria, or can introduce oxidative stress to the bacterial cells and be directly used as antimicrobial materials. These include metallic nanoparticles such as Ag, Cu, Au, etc. [17], nanocrystals of metal oxides such as CuO, Cu_2_O, Fe_3_O_4_, etc., [16,17] hybrid metal nanoparticle/2D materials [29,30], and hybrid organic–inorganic nanostructures [31,32]. Several recent reviews on inorganic antimicrobial nanomaterials can be found in Ref. [16,17].

The antimicrobial nanomaterial should at the very least be characterized by three important properties: efficiency in killing bacteria, toxicity to normal cells, and ease/cost of production. That is, an ideal antimicrobial nanomaterial should inhibit the maximum amount of bacteria in a short period of time, this efficacy should be universal to most pathogenic bacteria, the materials should be less or not toxic to normal cells, and the fabrication or synthesis of the materials should be cheap and easy. Table 1 summarizes the performance of some popular direct antimicrobial nanomaterials (inorganic) reported in the literature against two of the most widely studied bacteria, *E. coli* and *S. aureus*. Both the Ag and Cu_2_O nanoparticles (NPs) are found to be the most effective for killing *E. coli* and *S. aureus* as compared to other metal oxides. [33,34] It was reported that as little as 0.2 ppm of Ag NPs could inhibit 10^5^ CFU/mL of *E. coli* in 30 min, [33] while 50 μg/mL Cu_2_O octahedral nanocrystals could eradicate 2 × 10^7^ CFU/mL *E. coli* in 5 min in a 0.9% saline water dispersion [34]. CuO is also a very effective antimicrobial material, as shown in Table 1 [35]. Other oxides take one or two days to achieve a reasonable bactericidal effect. However, the materials with high antimicrobial performance, i.e., the NPs of Ag, Cu_2_O or CuO, were very toxic to human cells or in animal tests. Beer et al. showed that at 1.5 μg/mL total silver, the human pulmonary epithelial cells (A549) in vitro exposed to an Ag NP suspension containing a 69% silver ion fraction had a cell viability of 54% [36,37]. A recent study showed that the treatment with truncated octahedral Cu_2_O particles caused a significant reduction in animal size and lifespan in the nematode worm animal model *Caenorhabditis elegans* [38]. Siddiqui et al. demonstrated that when the A549 cells were exposed to CuO NPs with concentrations of 10, 25, and 50 µg/mL, the cell viabilities were 75%, 66%, and 48%, respectively [39]. In short-term oral exposure in rats, the CuO NPs induced changes in hematology parameters and liver damage, and histopathological alterations were observed in the bone marrow, stomach and liver, mainly consisting of an inflammatory response, ulceration, and degeneration [40]. Clearly, highly efficient single component antimicrobial materials are often accompanied by high toxicity due to the intrinsic antimicrobial mechanisms, which are not ideal for many applications.

In order to circumvent the above issue, different strategies, such as surface modification, composite materials or hybrid materials, have been used to reduce the toxicity of these antimicrobial materials [16,44]. For example, Tran et al. incorporated CuO NPs into cellulose and chitosan to form biocompatible antimicrobial composites [45]. They demonstrated that the composites were effective in reducing the growth of drug-resistant bacteria (e.g., approximately 3 log growth reduction in *E. coli* and 5 log growth reduction in *Streptococcus agalactiae*) with 298 nmol/mg CuO loading after 16 h incubation [45]. Many results showed that these strategies could not only reduce the toxicity significantly, but also induce an appreciable reduction in antimicrobial efficiency. Additionally, many of the proposed strategies involve multiple material preparation steps, which are sometimes not easy to synthesize or are not cost effective. In addition, in most antimicrobial studies, only two to four different bacteria were studied simultaneously, which makes the data comparison very difficult (for instance, to compare bactericidal efficiency from different sources). Thus, it is still important to discover new materials that can effectively inhibit a broad range (strains) of bacteria but with less toxicity and easy synthesis.

Here, we demonstrate that a mixture of cuprous oxide (Cu_2_O), iron oxide (Fe_2_O_3_) and copper iron oxide (CuFeO_2_) NPs (designated as Cu*_x_*Fe*_y_*O*_z_* NPs), synthesized through a one-step microwave-assisted hydrothermal method, had a very high antimicrobial activity (>5 log reduction within 4 h) against a broad range of Gram-positive and Gram-negative bacteria, including drug-resistant bacteria. The materials can also inhibit the growth of these bacteria suspended in culture media, and the inhibition depends strongly on the bacteria strains. The cytotoxicity tests against mouse fibroblast cells suggest that these NPs were less toxic to the mammalian cells at a concentration lower than 1 mg/mL. Collectively, the results suggest that the Cu*_x_*Fe*_y_*O*_z_* NPs are an excellent antimicrobial material, with a high bactericidal effect, low toxicity and easy synthesis.

## 2. Materials and Methods

*NP Synthesis and Characterizations:* The Cu*_x_*Fe*_y_*O*_z_* NPs were produced through a microwave-assisted hydrothermal synthesis (Monowave 400, Anton Paar, Graz, Austria). In a typical synthesis, 0.242 g Cu(NO_3_)_2_·3H_2_O (ACROS Organics, Fair Lawn, NJ, USA) and 0.404 g Fe(NO_3_)_3_·9H_2_O (Alfa Aesar, Haverhill, MA, USA) were thoroughly dissolved into 10 mL deionized (DI) water. First, 10 mL 1 M NaOH solution was added into the mixture dropwise with constant stirring. Next, 500 μL 37% formaldehyde (J.T. Baker, Allentown, PA, USA) was added into the mixture. A measure of 4 mL of the resulting solution was transferred into a 10 mL SiC reaction tube and kept in the microwave chamber at 200 °C for 2 h. After cooling down, the synthesized sample was centrifuged, washed with DI water for 5 iterations, and oven dried at 60 °C overnight. Morphologies of the samples were investigated by a field-emission scanning electron microscope (FESEM, FEI Inspect F, ThermoFisher Scientific, Hillsboro, OR, USA) equipped with an energy dispersive X-ray spectroscopy. High resolution scanning electron microscopy (SEM) analysis was carried out using a Hitachi SU9000 STEM/SEM (Hitachi, Chatsworth, CA, USA) at 30 kV, equipped with an Oxford solid state EDS detector (Oxford Instruments, Concord, MA, USA) to further investigate the morphologies and elemental composition of the Cu*_x_*Fe*_y_*O*_z_* NPs. The crystal structures of the prepared samples were characterized by an X-ray diffractometer (XRD; PANalytical X’Pert PRO MRD, Malvern Panalytical, Malvern, UK) with a Cu Kα source (*λ* = 1.5405980 Å) at 45 kV and 40 mA. The diffraction angle scanning range was 20° to 80° at an angular step of 0.02°. The Zeta potentials of the Cu*_x_*Fe*_y_*O*_z_* NPs were measured by a Malvern Zetasizer Nano ZS system (Malvern Panalytical, Westborough, MA, USA) at 25 °C. The dye degradation experiments were performed using methyl orange (MO) and methylene blue (MB) aqueous solutions with a concentration of 30 μM at room temperature. All experiments were performed with a concentration of the Cu*_x_*Fe*_y_*O*_z_* NPs at 0.5 mg/mL, and a fixed volume *V* = 20 mL of dye solutions. The reaction systems were kept in the dark with constant stirring. At each time interval (2, 4, 6, 8, 10 and 24 h), an aliquot sample was withdrawn and centrifuged at 12,000× *g* rpm to remove the NPs, and the concentration change of MO and MB in the remaining solution was investigated by UV-Vis.

*Antimicrobial activity tests:* The antimicrobial performance of the samples was tested on various bacterial strains (see Table S1 of SI). The bacterial stocks were activated in fresh liquid media (trypticase soy broth (TSB) for *E. coli* B and *S. aureus*; Luria Bertani broth (LB) for *E. coli* O157:H7, *Klebsiella pneumoniae* 4/484, *K. pneumoniae* ATCC-BAA-2472, *Listeria monocytogenes*, *Salmonella enterica* serovar Typhimurium ATCC-700408, and *Shigella flexneri*) overnight at 37 °C with shaking (250 rpm). *H. pylori* cells were routinely grown on blood agar (BA) plates under microaerobic conditions (5% O_2_, 5% CO_2_, 90% N_2_). For the antimicrobial tests with bacterial cells in phosphate buffered saline (PBS), bacterial cells grown in the media described above were harvested by centrifugation at 4000× *g* rpm for 5 min, washed twice with PBS, and finally re-suspended in PBS. For *E. coli* B and *S. aureus* tests, 1 mg of Cu*_x_*Fe*_y_*O*_z_* NPs was directly suspended in the bacteria suspension. For other bacterial strains, the cells were first standardized to an OD_600_ of 2 in PBS (~10^9^ to 2 × 10^9^ CFU/mL depending upon strains), then 0.25 mL of each cell suspension was diluted with either 0.25 mL of PBS as controls or 0.25 mL of 2 mg/mL Cu*_x_*Fe*_y_*O*_z_* NPs. The mixtures of bacteria and Cu*_x_*Fe*_y_*O*_z_* NPs were incubated at 37 °C with shaking (200 rpm). Aliquots were withdrawn at various time intervals (15, 30, 60, 120, and 240 min, respectively), and 10-fold serially diluted in PBS. The dilutions were plated on media plates and incubated at 37 °C for 12 to 16 h. *H. pylori* cell dilutions were incubated on BA plates for 3 to 5 days under microaerobic conditions. All antimicrobial activity tests were done in triplicate.

*Bacterial growth inhibition tests:* For growth inhibition tests, bacterial cells were inoculated into fresh media with Cu*_x_*Fe*_y_*O*_z_* NP suspensions. For *E. coli* B and *S. aureus* tests, 10 mg Cu*_x_*Fe*_y_*O*_z_* NPs were briefly suspended in 0.1 mL PBS first, then added into 9.9 mL TSB (final NPs concentration was 1 mg/mL). Next, a 10 μL bacteria suspension was inoculated into the media–NPs mixture and kept at 37 °C for 12 to 16 h with shaking (250 rpm). For the *K. pneumoniae* BAA-2472, *S.* Typhimurium 700408, and *S. flexneri* experiments, a 2 μL bacteria suspension (corresponding to approximately 2 × 10^6^ cells) was inoculated in 500 μL TSB with 1 mg/mL Cu*_x_*Fe*_y_*O*_z_* NPs. For *H. pylori*, cells were grown on BA plates, harvested, and resuspended in 100 μL of brain–heart infusion with 0.4% β-cyclodextrin (BHI-βc) to a final concentration of approximately 10^7^ cells, and were then inoculated into 2 mL BHI-βc supplemented with 0.1 mL 20 mg/mL Cu*_x_*Fe*_y_*O*_z_* NPs in PBS (final NPs concentration was 1 mg/mL). These bacteria–media–NP mixtures were then incubated at 37 °C for 18 h under aerobic (all strains except *H. pylori*) or microaerobic (for *H. pylori*) conditions with constant rocking or shaking. After co-incubating with NPs in media, an aliquot amount of the mixture was withdrawn from each sample and 10-fold diluted in PBS. The dilutions were plated onto agar plates and incubated at 37 °C as described above. Control groups were performed with an equal volume of PBS to replace the Cu*_x_*Fe*_y_*O*_z_* NP suspension. All bacterial growth inhibition tests were done in triplicate.

*Cytotoxic activity tests:* The cytocompatibility test of the Cu*_x_*Fe*_y_*O*_z_* NPs was performed using a standard WST-8 dye-based assay in accordance with the manufacturer’s recommendations (Sigma-Aldrich). During the experiment, mouse fibroblast cells were grown in a 75 cm^2^ T-flask and incubated in a humidified CO_2_ incubator for 6–7 days at 37 °C until 80–90% confluent. Thereafter, cells were detached using 0.5% trypsin EDTA, counted via trypan blue assay (0.4%) (EVE Automatic cell Counter) and 100 µL of 5000 cells/mL were seeded in a 96 well plate. The plate was kept for 24 h at 37 °C in a CO_2_ incubator to allow cells to fully grow and attach to the surface. In parallel, a 1 mg/mL or a 10 mg/mL Cu*_x_*Fe*_y_*O*_z_* solution was prepared in Dulbecco’s Modified Eagle’s Medium (DMEM). The cells were allowed to grow in the well plate at 37 °C for 24 h. After 24 h, the medium was replaced with the prepared medium containing 1 mg/mL or 10 mg/mL Cu*_x_*Fe*_y_*O*_z_.* The cells grown in the well plate in the absence of Cu*_x_*Fe*_y_*O*_z_* NPs were used as a control group (*n* = 8 for each condition). After 24 h of the exposure, 10 µL of WST-8 dye was added to each of the wells containing cells. Viable cells converted the WST-8 dye into a yellow-colored formazan product. The DMEM medium was used as a blank to baseline the background. The absorbance of formazan was measured at 450 nm with a BioTek spectrophotometer as a measure of cell viability. The relative change in cell viability was calculated considering a 100% control viability. All cytotoxic activity tests were done in triplicate.

*Statistical analysis*: all results shown are means and standard deviations for experiments done in triplicate. When required, statistical analysis was done using Student’s *t*-test.

## 3. Results

### 3.1. Synthesis and Characterizations of Cu_x_Fe_y_O_z_ NPs

**Figure 1:**
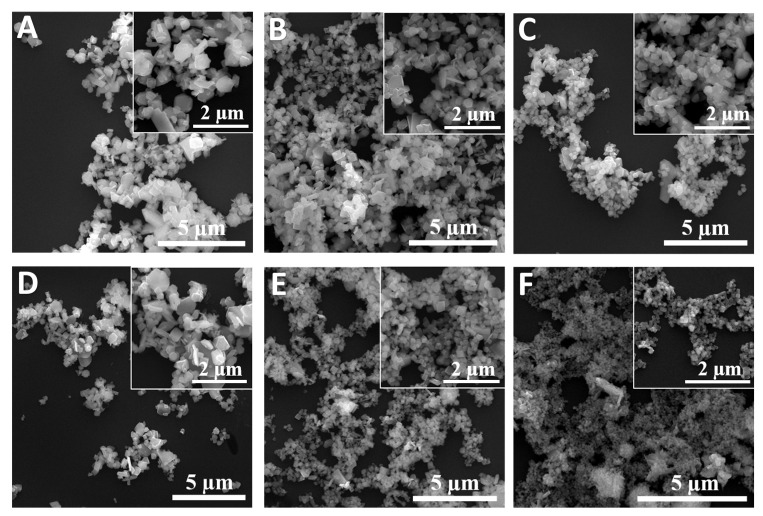
Representative SEM images of Cu*_x_*Fe*_y_*O*_z_* NPs designated by CH_2_O addition in synthesis. (**A**) S200; (**B**) S250; (**C**) S300; (**D**) S350; (**E**) S400; and (**F**) S500.

**Figure 2:**
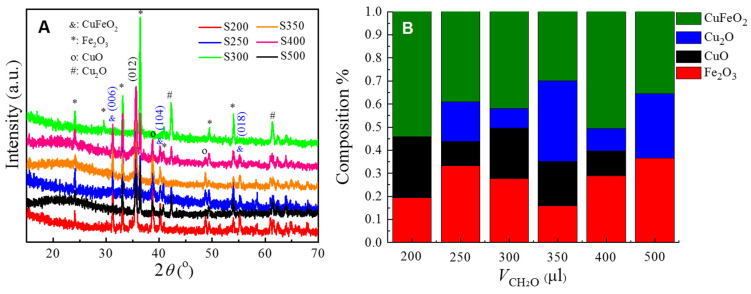
(**A**) The XRD patterns of the six samples S200 to S500. The diffraction peaks from CuFeO_2_, CuO, Cu_2_O, and Fe_2_O_3_ are indicated in the plot. (**B**) The relative compositions of CuFeO_2_, CuO, Cu_2_O, and Fe_2_O_3_ in each samples extracted from the Rietveld program.

**Figure 3:**
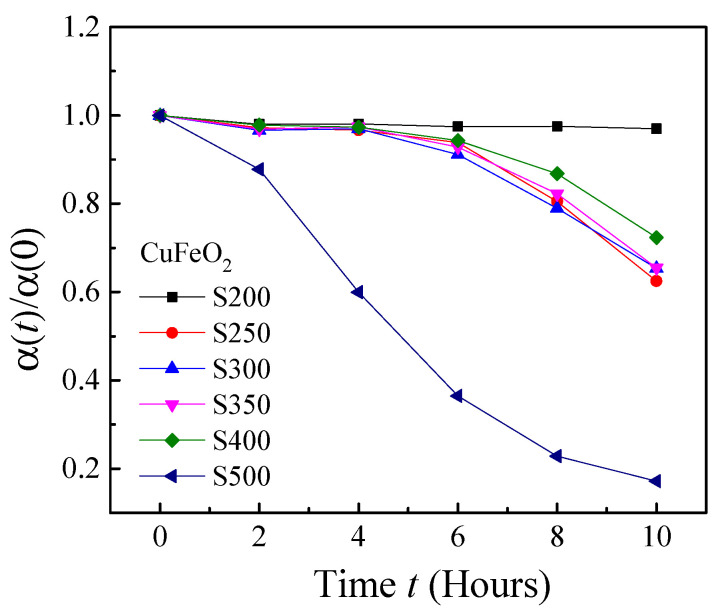
Catalytic activity of different Cu*_x_*Fe*_y_*O*_z_* NP samples: remaining concentrations of methyl orange after exposure to Cu*_x_*Fe*_y_*O*_z_* NP samples. Dark conditions were maintained throughout the entire process.

During the microwave hydrothermal synthesis, we kept the amounts of the Cu(NO_3_)_2_·3H_2_O, Fe(NO_3_)_3_·9H_2_O and NaOH the same, and systematically changed the volume of 37% formaldehyde from 200 μL to 500 μL to obtain different Cu*_x_*Fe*_y_*O*_z_* NPs. We denote the name of the samples as S200, S250, …, S500, which correspond to the volumes of formaldehyde 200 μL, 250 μL, …, and 500 μL, respectively. Figure 1 shows the representative SEM images of the resulting Cu*_x_*Fe*_y_*O*_z_* NPs. Despite the detailed synthesis conditions, the Cu*_x_*Fe*_y_*O*_z_* NPs consist of NPs of different sizes and shapes, including rod-shaped, plate-like-shaped, and other irregular shapes, and the average size of the NPs became smaller and smaller when more and more formaldehyde was added. In fact, based on the scanning electron microscopy (SEM) images and the dynamic light scattering (DLS) measurements, the average size of the NPs decreased monotonically as a function of the amount of formaldehyde added (see Figure S1 of Supporting Information (SI)) [46]. The crystallinity of the NPs was characterized by X-ray diffraction (XRD) as shown in Figure 2A. All 6 samples are a mixture of multiple oxides, as revealed by the XRD pattern—the 2θ peaks at 24.1°, 29.6°, 33.1°, 40.8°. 49.4° and 54.0° correspond to the (012), (220), (104), (113), (024), and (116) crystal planes of *α*-Fe_2_O_3_ (indicated by “*” in Figure 2A), the XRD peaks at 38.7° and 48.7° are due to the (200) and (202) crystal planes of CuO (indicated by “o”), the peaks at 42.2° and 61.3° are the (200) and (220) crystal planes of Cu_2_O (indicated by “&”), and the peaks at 31.2°, 35.6°, 40.2°, and 55.1° are from the (006), (012), (104), and (018) crystal planes of CuFeO_2_. After careful analysis of the XRD peak positions, one finds that only the samples S200 and S500 are a mixture of three oxides, i.e., CuFeO_2_, CuO, and Fe_2_O_3_ for S200, and CuFeO_2_, Cu_2_O, and Fe_2_O_3_ for S500. The other four samples are a mixture of all four different oxides, i.e., CuFeO_2_, CuO, Cu_2_O, and Fe_2_O_3_. The formation of the Cu*_x_*Fe*_y_*O*_z_* NPs could be the intermediate product towards the formation of single-phase CuFeO_2_ NPs, since the reaction time for the synthesis was very short (2 h). For example, the rhombohedral CuFeO_2_ crystals reported by Qiu et al. were formed under the prolonged hydrothermal reaction times of 24 h, 48 h, and 64 h, respectively [47], while Abdelhamid et al. spent 24 h to obtain CuFeO_2_ NPs capped with glycerol [48]. The XRD patterns can also be used to roughly estimate the relative composition ratio of the four oxides according to a semiquantitative analysis utilizing the Rietveld program from the FullProf software, [49,50,51,52] and the results are shown in Figure 2B. For sample S200, the relative compositions of CuFeO_2_, CuO, and Fe_2_O_3_ are 54%, 26%, and 20%, respectively, while for S500, the ratios for CuFeO_2_, Cu_2_O, and Fe_2_O_3_ are 36%, 28%, and 36%, respectively. Thus, the sample S500 has an almost equal amount of the three oxides. Among all six samples, the sample S350 has the highest composition of Cu_2_O, sample S200 has the highest composition of CuFeO_2_ and CuO, and sample S500 has the most Fe_2_O_3_ in its composition. The catalytic performance of Cu*_x_*Fe*_y_*O*_z_* NPs was characterized by a dye degradation against methyl orange (MO) under the dark condition through a UV-Vis spectroscopy measurement, and the normalized optical absorbance peak *α* at the characteristic wavelength *λ*= 464 nm of MO solution against the decay time *t* is plotted in Figure 3. The sample S200 shows almost no decay of MO, while S500 demonstrates the highest degradation, i.e., almost 80% of MO was decomposed after 10 h. This indicates that the sample S500 exhibits the highest catalytic activity among all the Cu*_x_*Fe*_y_*O*_z_* NPs synthesized. Based on previous literature, the high catalytic performance of a material usually links to a high antimicrobial activity [53,54]. Therefore, in the following, we will concentrate on the sample S500.

**Figure 4:**
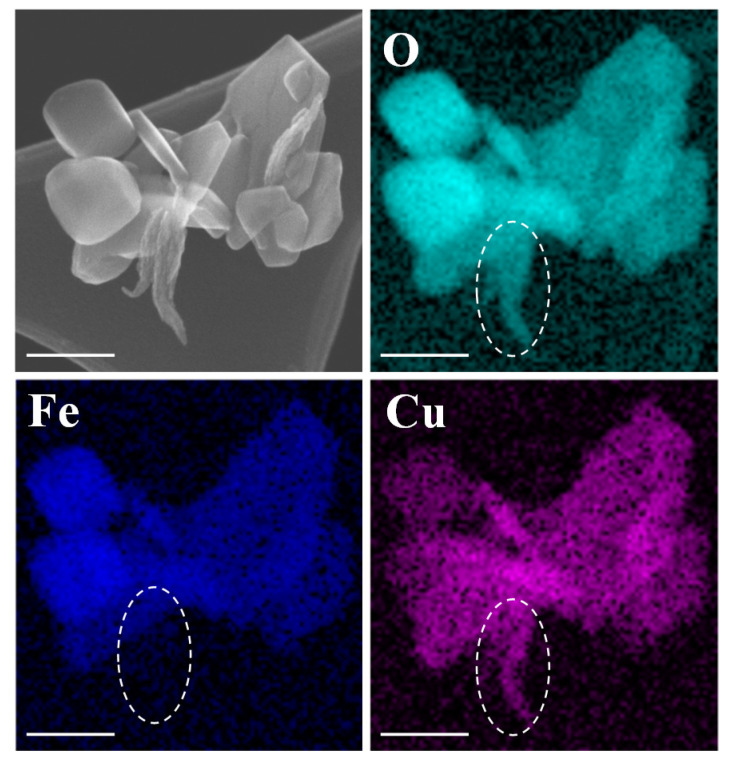
EDS mapping (elements of O, Fe, and Cu) of a Cu*_x_*Fe*_y_*O*_z_* (S500) NP cluster. The scale bars represent 100 nm. The dashed-oval outlines the Cu_2_O NPs.

The as-synthesized sample S500 comprises various shapes of particles, such as cubic, flake, and irregular shapes (Figure 1F), with a mean size of 100 ± 20 nm in diameter. The higher magnification scanning electron micrograph (SEM) and the energy dispersive X-ray spectroscopic (EDS) mapping (Figure 4) show that oxygen is uniformly distributed on all structures, but the Fe and Cu atoms are not distributed evenly. There are greater Fe contents in the cubic NPs, while the strip structures are mainly composed of Cu contents. According to the XRD analysis in Figure 2, the strip structures in Figure 4 could mainly be the Cu_2_O, while the other shaped NPs are Fe_2_O_3_ and CuFeO_2_. In addition, the sample S500 shows strong dye degradation activities against the cationic dye, MO (~90% reduction in 24 h), but is less effective on the anionic dye, methylene blue (MB) (~8% reduction in 24 h (see Figures S2 and S3 in SI)) when tested with 0.5 mg/mL NP suspensions in the dark after a 24 h treatment. This could be attributed to the fact that the decomposition of MB mainly occurs through reduction reactions [55], while the decomposition of MO is mainly due to oxidation reactions [56]. That is, the results suggest that the as-synthesized Cu*_x_*Fe*_y_*O*_z_* NPs are efficient oxidizing agents. Similar properties of other Cu*_x_*Fe*_y_*O*_z_* NP samples are summarized in Table S2.

### 3.2. Antimicrobal Characterizations of S500

**Figure 5:**
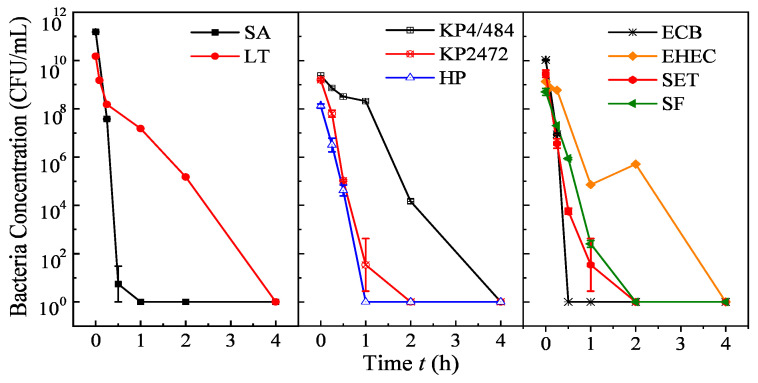
Antimicrobial tests of Cu*_x_*Fe*_y_*O*_z_* NPs (1 mg/mL in PBS) against various pathogenic bacteria. The acronyms for bacteria are listed in Table 2. Results represent 3 independent challenge experiments (2 for KP4/484) and are expressed as mean and standard deviation of bacterial concentration (in CFU/mL).

The outstanding MO oxidizing activity suggests that the S500 should have good antimicrobial properties. To test this hypothesis, antimicrobial tests were carried out using nine important pathogenic bacterial strains, including Gram-positive species such as *S. aureus* Rosenbach ATCC-6538 and *L. monocytogenes,* and Gram-negative species, such as enterohemorragic *E. coli* (EHEC) strain O157:H7, *K. pneumoniae* strain 4/484 and strain ATCC-BAA-1472, *S.* Typhimurium ATCC-700408, *H. pylori* X47, and *S. flexneri* strain 2457T; among those are the multidrug-resistant (MDR) *K. pneumoniae* 4/484 and BAA-2472, and *S.* Typhimurium 700408. The nonpathogenic *E. coli* strain B was used as a reference (the details on the bacteria strains can be found in Table S1 of SI). The final Cu*_x_*Fe*_y_*O*_z_* NP (S500) concentration was 1 mg/mL throughout all experiments. To ensure that the sample S500 shows better antimicrobial activity, antimicrobial tests against *E. coli* B for both S200 and S500 with different concentrations were conducted. The results are shown in Figure S4 of SI, which confirms that sample S500 has better antibacterial activity. Figure 5 shows the plots of the time-dependent bacterial survival loads, while Table 2 summarizes the significant bacterial killing efficiency observed for different bacterial strains. The Cu*_x_*Fe*_y_*O*_z_* NPs (S500) show high antimicrobial activity against almost all the bacterial strains tested. First, the Cu*_x_*Fe*_y_*O*_z_* NPs can kill both Gram-negative and Gram-positive bacteria very effectively. The results showed a more than 9 log reduction in 0.25 h (15 min) for *E. coli* B and a 10 log reduction within 1 h for *S. aureus*. For other bacteria, such as *E. coli* O157:H7 and *L. monocytogenes*, a more than 7 log reduction was observed upon 4 h of Cu*_x_*Fe*_y_*O*_z_* NPs exposure. The results indicated that these nanoparticles could effectively kill the bacterial cells within a very short exposure term. Compared to the best antimicrobial activities of other inorganic nanostructures shown in Table 1, only Ag NPs or Cu_2_O NPs can achieve similar or better results. However, both Ag and Cu_2_O NPs are highly toxic to host cell lines, while the Cu*_x_*Fe*_y_*O*_z_* NPs are not (see toxicity test later). In addition, the Cu*_x_*Fe*_y_*O*_z_* NPs also exhibited rapid and effective antimicrobial activities against highly infectious drug-resistant strains—the biocidal efficiencies of the Cu*_x_*Fe*_y_*O*_z_* NPs against MDR *K. pneumoniae* 4/484, BAA-2472, and *S.* typhimurium 700408 showed an 8.4 and 6.9 log reduction, respectively, in 4 h, and a 7.2 log reduction in 2 h, respectively.

**Figure 6:**
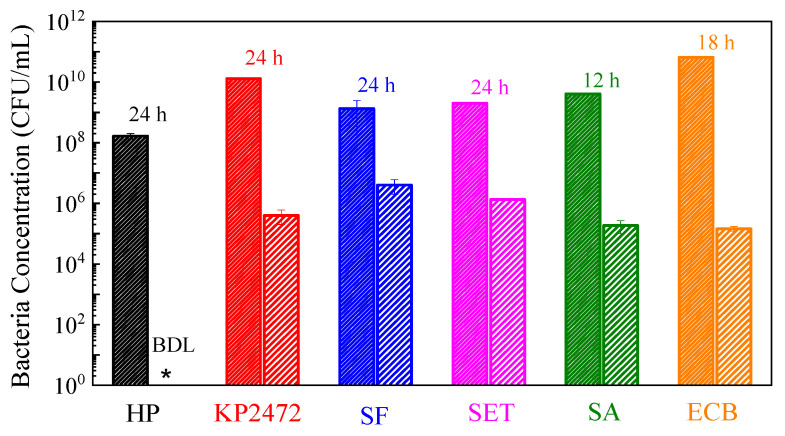
Bacterial inhibition tests: co-culturing bacteria with 1 mg/mL Cu*_x_*Fe*_y_*O*_z_* NPs in corresponding growth media. The results represent 3 independent growth experiments and are expressed as mean and standard deviation of CFU/mL (at the time indicated above each column). BDL: below detection limit (<200 CFU/mL). For each bacterial species, the bacterial concentration after Cu*_x_*Fe*_y_*O*_z_* treatment is significantly lower compared to the bacterial concentration obtained without treatment (*p* < 0.01, Student’s *t* test).

The high antimicrobial activity of the S500 was also demonstrated upon bacterial incubation under favorable culture conditions (bacteriostatic effect) when cells were incubated with the inhibitor from the start of culture. Both the Cu*_x_*Fe*_y_*O*_z_* NPs and bacteria were incubated in adequate fresh broth culture media, and bacterial growth was monitored by CFU counting after serial dilutions. The results are summarized in Figure 6. These NPs inhibited the growth of the bacteria under favorable growth conditions. As such, *H. pylori* growth was completely inhibited after 24 h co-culturing in the BHI-βc media; in contrast, *H. pylori* achieved a concentration as high as 10^8^ CFU/mL in the control group. For the test against MDR *K. pneumoniae* BAA-2472, a 4 log reduction was obtained after 24 h growth. Similar results were obtained for *S flexneri*, *S. aureus* and *E. coli—*these bacteria were all subject to inhibition by Cu*_x_*Fe*_y_*O*_z_* NPs. It is expected that, since this is a competition between the cell growth and cell killing, the number of bacterial cells that survived the bacteriostatic effect may be much greater than those in the antimicrobial tests. The tests further suggest that the S500s are promising antimicrobial agents.

**Figure 7:**
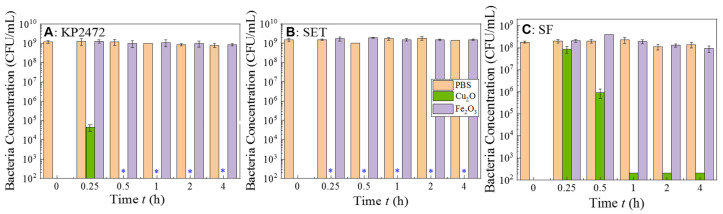
Antimicrobial tests of 1 mg/mL control Fe_2_O_3_ and Cu_2_O NPs against various pathogenic bacteria (**A**) *K. pneumoniae* (KP2472), (**B**) *S*. Typhimurium (SET), and (**C**) *S. flexneri* (SF). These tests were carried out in the dark. The results represent 3 independent growth experiments and are expressed as mean and standard deviation of CFU/mL. The treatment leading to a significant reduction in bacterial concentration is indicated by a blue asterisk (*p* < 0.01 Student’s *t* test).

The antimicrobial mechanism of the Cu*_x_*Fe*_y_*O*_z_* NPs (S500) is not yet well understood. According to the composition analysis shown in Figure 2, there are three kinds of NPs in the mixture: Cu_2_O, CuFeO_2_, and Fe_2_O_3._ Cu_2_O NPs are well-known antimicrobial nanomaterials [34] and recent reports show that CuFeO_2_ NPs can kill viruses [47] and fungi [48]. The Fe_2_O_3_ NPs are a photocatalytic material and they can only show antimicrobial activity when illuminated by light with energy higher than the energy band gap [28]. Thus, we hypothesize that the antimicrobial properties might mostly originate from the Cu_2_O and CuFeO_2_. For these two materials, the antimicrobial properties are attributed to a Cu^+^ species in the compounds [47,48]. To gain a better insight, we have also synthesized two control NPs (the Fe_2_O_3_ and Cu_2_O NPs) under similar conditions, and their characterizations are presented in Section S5 of SI. Their antimicrobial activities against KP2472, SET and SF are shown in Figure 7. The Fe_2_O_3_ NPs alone do not demonstrate any antimicrobial effect, while the biocidal efficiency values of the Cu_2_O NPs for KP2472, SET and SF are 9 log, 9 log and 8 log reductions in 30 min, 15 min, and 60 min, respectively. This shows that the Cu_2_O NPs are effective antimicrobial agents, which is consistent with results reported in the literature. [34,57,58] Compared to the antimicrobial activity against corresponding bacteria for S500, the Cu_2_O NPs are more effective. Since the S500 is a mixture of Cu_2_O, CuFeO_2_ and Fe_2_O_3_ NPs, such a result is expected, which means that the Cu_2_O NPs in S500 could play a major role in its antimicrobial activity.

**Figure 8:**
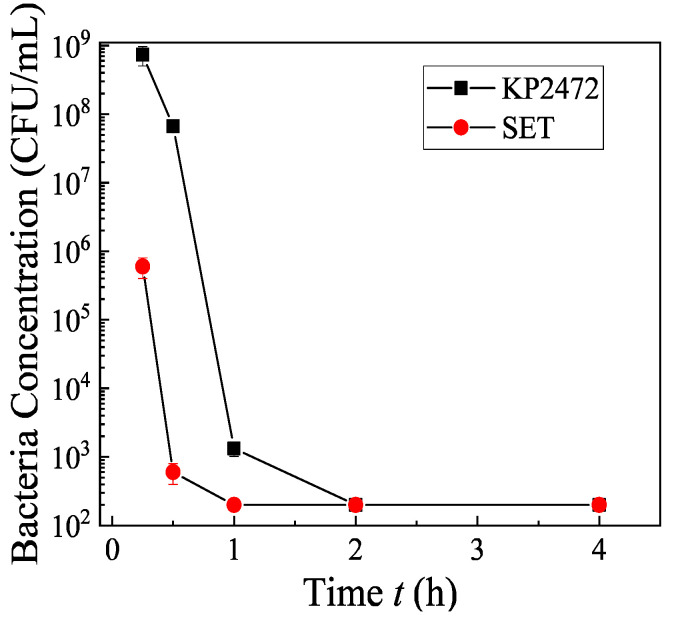
Antimicrobial tests of a mixture of the control Fe_2_O_3_ and Cu_2_O NPs against KP2472 and SET in a dark environment. The results represent 3 independent growth experiments and are expressed as mean and standard deviation of CFU/mL.

The role of CuFeO_2_ NPs is difficult to determine since we could not directly synthesize pure CuFeO_2_ NPs via the microwave-assisted hydrothermal method. Based on previous reports [47,48], the CuFeO_2_ NPs should also have a strong antimicrobal effect. In fact, according to a comparison study by Antonoglou et al., the CuFeO_2_ NPs exhibited less antimicrobial activity than the Cu_2_O NPs, but their cytotoxity was also lower [59]. Since the S500 sample has 36% CuFeO_2_, 0% CuO, 28% Cu_2_O and 36% Fe_2_O_3_ (Figure 2b), one could use a control mixture of Fe_2_O_3_ and Cu_2_O NPs (their crystalline properties are shown in Figure S5) to indirectly explore the effect of CuFeO_2_ NPs. The control mixture had a mass ratio of Cu_2_O NPs:Fe_2_O_3_ NPs = 28:36, with a total NP concentration of 0.64 mg/mL (i.e., mimicking the S500 without CuFeO_2_) against KP2472 and SET, and the results are shown in Figure 8. The antimicrobial activity values of the control mixture for KP2472 and SET are 9 log and 6 log reductions in 2 h and 1 h, respectively. Clearly, the control mixture also has a higher antimicrobial activity as compared to that of sample S500, which indicates that the sample S500 is not a simple mixture of Cu_2_O, CuFeO_2_, and Fe_2_O_3_ NPs. In fact, based on the SEM image shown in Figure 4, the mixture of Cu_2_O, CuFeO_2_ and Fe_2_O_3_ NPs in sample S500 forms super clusters, and the three different NPs physically join together to form complicated hetero-interfaces. These hetero-interfaces could form semiconductor junctions due to the energy band realignment, and change the electron structures of the materials so that the corresponding catalytic activity will be greatly changed compared to that of pure Cu_2_O or Fe_2_O_3_ NPs. The control mixture of Cu_2_O and Fe_2_O_3_ NPs is a simple blend, and they act separately on bacteria.

**Figure 9:**
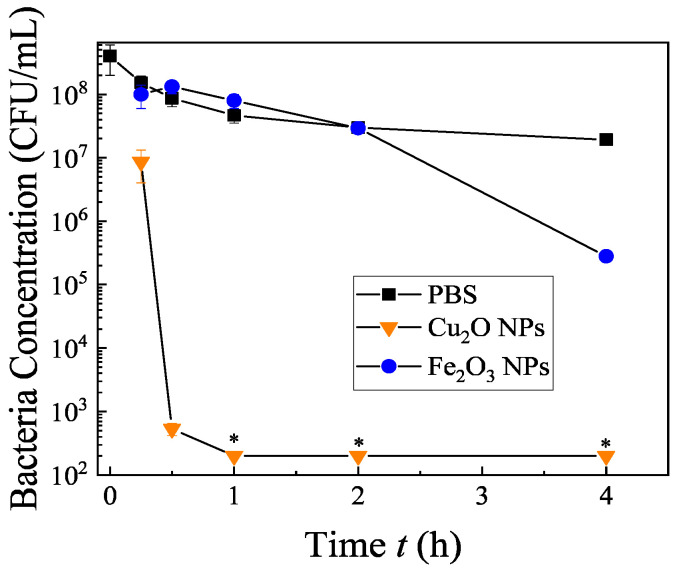
Bacteria inhibition tests for 1 mg/mL control Fe_2_O_3_ and Cu_2_O NPs in corresponding growth media. “*”: ND/below detection limit (200 CFU/mL). These tests were carried out in a dark environment. The results represent 3 independent inhibition experiments and are expressed as mean and standard deviation of CFU/mL.

The bacteria growth inhibition tests of the control Cu_2_O and Fe_2_O_3_ NPs (1 mg/mL) were performed against HP, and the results are shown in Figure 9. Similar conclusions can be drawn for the antimicrobial tests: the Fe_2_O_3_ NPs do not inhibit the bacteria growth, while the Cu_2_O NPs inhibit the growth significantly (i.e., 9 log inhibition for 1 h).

It is well-known that copper ions are capable of killing microorganisms effectively by denaturation (enzyme, protein, etc.) or oxidation mechanisms [60,61]. It was reported that Cu_2_O nanocrystals could generate reactive oxygen species (ROS) through biochemical processes [62]. In general, during these biochemical processes, as a redox-active transition metal, Cu can cycle between two redox states—oxidized cupric and reduced cuprous states. It is suggested that Cu can react with endogenous H_2_O_2_ to generate hydroxyl radicals in a process analogous to the Fenton reaction, and it can also catalyze the transfer of electrons from a donor biomolecule to an acceptor such as O_2_ to generate O_2_^−•^ or hydroxyl radicals (·OH) [46,62]. These ROS are toxic to bacterial cells, which can disrupt specific microbial processes. The direct effects of ROS on bacterial cells are mediated by the increasing production of ROS, which can lead to the oxidative damage of the cellular compounds [63]. Studies show that the ROS could damage cell membranes, subsequently causing cytoplasmic metabolites to leak and ion gradients to collapse, leading to cell death [35,64]. Furthermore, the ROS can destroy key macromolecules, such as the iron–sulfur cluster components of enzymes, to disintegrate and oxidize amino acid residues within proteins, generate lipid peroxides, and damage DNA [64,65]. On the other hand, Antonoglou et al. showed that the CuFeO_2_ NPs had a better biocompaibility for DNA and were less effective in protein denaturing [59]. The addition or clustering of CuFeO_2_ and Fe_2_O_3_ NPs with Cu_2_O NPs in S500 significantly slows down the bacteria inhibition process.

### 3.3. Cytotoxicity of the Cu_x_Fe_y_O_z_ NP Sample S500

**Figure 10:**
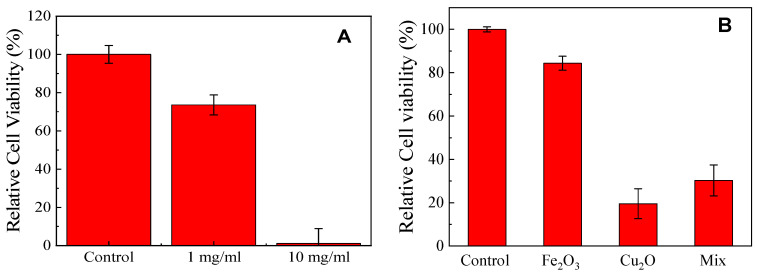
(**A**) Cytotoxicity of the Cu*_x_*Fe*_y_*O*_z_* NPs against mouse fibroblast cells: cell viability after cells exposed to Cu*_x_*Fe*_y_*O*_z_* NPs at 0 mg/mL (control), 1 mg/mL, and 10 mg/mL for 24 h. (**B**) Cytotoxicity of the control NPs against mouse fibroblast cells: cell viability after cells exposed to NPs at 1 mg/mL for 24 h. The results represent 3 independent experiments and are expressed as mean and standard deviations of relative cell viability (compared to control).

It is also well-known that a high concentration of ROS and Cu ions can also trigger damage on mammalian cells. Thus, the cytotoxicity of the Cu*_x_*Fe*_y_*O*_z_* NPs (S500) against mammalian cells needs to be explored. The cytotoxicity test of the Cu*_x_*Fe*_y_*O*_z_* NPs was performed by the exposure of mouse fibroblast cells to different concentrations of Cu*_x_*Fe*_y_*O*_z_* NPs at 37 °C for 24 h. As shown in Figure 10A, fibroblasts treated with 10 mg/mL Cu*_x_*Fe*_y_*O*_z_* NPs could only retain less than 10% cell viability. However, when treated with 1 mg/mL Cu*_x_*Fe*_y_*O*_z_* NPs (which was the concentration used for antimicrobial activity tests, see Figure 5 and Figure 6 and Table 2), about 74% of the fibroblasts remained viable. Results for control NPs (1 mg/mL for Cu_2_O and Fe_2_O_3_ NPs, and 0.64 mg/mL for the Cu_2_O and Fe_2_O_3_ NP mixture) are shown in Figure 10B. For Cu_2_O NPs, the number of viable cells rapidly decreased to 20 ± 7%; compared to Cu_2_O NPs, the cell viability was moderately affected by the presence of Fe_2_O_3_ NPs. The mixed Cu_2_O and Fe_2_O_3_ NPs, however, exhibited a high degree of cytotoxicity (the viability goes down to 30 ± 7%). Thus, it becomes increasingly evident that the cytotoxic effects elicited on the fibroblast cells are primarily due to Cu_2_O NPs. Clearly, the cytotoxicity result for Cu*_x_*Fe*_y_*O*_z_* NPs (S500) is comparable to that of the control Fe_2_O_3_ NPs. In comparison to other high-efficiency antimicrobial inorganic materials, for example the CuO NPs, it was reported that at a concentration of 80 μg/mL, the CuO NPs were toxic to lung cells and could also cause DNA damage to the cells [66]. Thus, many efforts have been made to cap the CuO or Cu_2_O NPs with polymers or ligands to reduce the toxicity. However, such a coating could also decrease the antimicrobial activity. Our results suggest that the Cu*_x_*Fe*_y_*O*_z_* NPs (S500) are toxic to the mouse fibroblasts only when used at a very high concentration (10 mg/mL). The cyctotoxity of the Cu*_x_*Fe*_y_*O*_z_* NPs at high concentrations also indirectly implies that the Cu^+^ from the mixture could play an important role in antimicrobial activity. However, at a lower concentration of 1 mg/mL, they are much less toxic. At this concentration (1 mg/mL), Cu*_x_*Fe*_y_*O*_z_* NPs are found to have highly efficient antimicrobial activities against a wide range of microorganisms. As such, the low toxicity to mammalian cells combined with the strong antimicrobial activity against some of the most important human pathogens suggest that the Cu*_x_*Fe*_y_*O*_z_* NPs inhibitory agents could be broadly applicable, such as for water treatment applications, wound treatments, or when used as coatings for medical devices.

## 4. Conclusions

In summary, we have synthesized mixed composition Cu*_x_*Fe*_y_*O*_z_* NPs using a facile one-step microwave assisted hydrothermal synthesis. The resulting samples were a mixture of CuFeO_2_, CuO, Cu_2_O and Fe_2_O_3_, and all of them show catalytic activity against MO solutions. The sample S500 showed the best MO-degradation among all of the samples, and was selected to conduct the detailed antimicrobial study. The EDS and XRD results confirmed that the S500 sample had a composition of Cu_2_O, Fe_2_O_3_ and CuFeO_2_. The Cu*_x_*Fe*_y_*O*_z_* NPs also showed highly effective antimicrobial activity (>5-fold log reduction within 4 h) against different Gram-positive and Gram-negative bacteria, including three drug-resistant strains. These antimicrobial activities are among the highest reported in the literature. Two of the inhibited bacteria strains are within the highly recalcitrant ESKAPE group of pathogenic bacteria, the bacterial group causing the majority of nosocomial infections throughout the world [67]. Moreover, *S. aureus, K. pneumoniae* and *E. coli* strains cause nearly 30% of all nosocomial infections in the USA [68]. When co-culturing the NPs with bacteria in growth media, the NPs can also eliminate bacteria growth. The toxicity test showed that the mouse fibroblast cell viability was about 74% when exposed to 1 mg/mL Cu*_x_*Fe*_y_*O*_z_* NPs for 24 h, as compared to the 69% viability of 1.5 μg/mL Ag NPs [36,37] and the 75, 66 and 48% viability of 10, 25, and 50 µg/mL CuO NPs [39]. Thus, Cu*_x_*Fe*_y_*O*_z_* NPs have low toxicity and could selectively target bacteria, which is a great advantage compared to other inorganic antimicrobial materials. Furthermore, when compared to silver NPs, the Cu*_x_*Fe*_y_*O*_z_* NPs are inexpensive and chemically stable. Evidently, the Cu*_x_*Fe*_y_*O*_z_* NPs are an excellent antimicrobial material. However, there are still many unanswered questions for this study, as follows: (1) Why do the Cu*_x_*Fe*_y_*O*_z_* NPs have a high antimicrobial efficacy but low toxicity, while a mixture of Cu_2_O and Fe_2_O_3_ NPs is still highly toxic? To answer this question, one needs to have a good understanding of the detailed microstructure of the Cu*_x_*Fe*_y_*O*_z_* NPs, as well as of how the particle interacts with the bacteria cells. (2) What is (are) the mechanism(s) for the bactericidal effect? Is it really caused by Cu^+^ species or by the production of reactive oxygen species? Or other mechanisms? (3) Is the S500 sample the best antimicrobial material out of similar fabrication strategies? We have only done a very limited investigation on Cu*_x_*Fe*_y_*O*_z_* NP synthesis by varying the amount of formaldehyde in the reagents. It is unknown what would happen if the concentrations of the Cu(NO_3_)_2_·3H_2_O, Fe(NO_3_)_3_·9H_2_O and NaOH were changed. Thus, a more systematic study on synthesis conditions may find a better antimicrobial material. Nevertheless, our current studies do show that the synthesis method of Cu*_x_*Fe*_y_*O*_z_* NPs is simple. The raw materials for synthesis are abundant, and these Cu*_x_*Fe*_y_*O*_z_* NPs are an excellent candidate for antimicrobial applications, such as antimicrobial cream, spray, and coating, as well as assays for disinfections or water treatment.

## Figures and Tables

**Table 1:** Summary of common inorganic antimicrobial materials and their best antimicrobial activity reported in the literature.

Material	Bacterial Strain	Reduction	Test Time	Reference
Silver (Ag)	*E. coli/S. aureus*	5 log	0.5 h/1.5 h	[33]
Copper oxide (CuO)	*E. coli*	3 log	3 h	[35]
Cuprous oxide (Cu_2_O)	*E. coli*	7 log	5 min	[34]
Nitric oxide (NO)	*E. coli/A. baumannii/S. aureus*	8 log	24 h	[41]
Zinc oxide (ZnO)	*E. coli*	6 log	48 h	[42]
Copper (Cu)	*E. coli/S. aureus*	4 log	24 h	[43]

**Table 2:** Antimicrobial activity of the Cu*_x_*Fe*_y_*O*_z_* NPs (bacterial cells were suspended in 1X PBS).

Bacteria Strain	Log Reduction	Time/h
*E. coli* B (ECB) (−)	>9.0	0.25
*S. aureus* (SA) (+)	>10.2	1
*K. pneumoniae* 4/484 (KP4/484) (−)	>8.4	4
*E. coli* O157:H7 (EHEC) (−)	>7.1	4
*Listeria* (LT) (+)	>9.2	4
*K. pneumoniae* BAA-2472 (KP2472) (−)	>6.9	2
*S.* Typhimurium 700408 (SET) (−)	>7.2	2
*H. pylori* X47 (HP) (−)	>5.8	1
*S. flexneri* 2457T (SF) (−)	>6.4	2

+: Gram-positive bacteria; −: Gram-negative bacteria.

## Supplementary Materials

The following are available online at https://www.mdpi.com/2079-4991/10/11/2294/s1: Table S1: Bacterial strains used in this study. Table S2: Summary of the properties of Cu*_x_*Fe*_y_*O*_z_* NP samples. Figure S1: Size comparison between SEM data and dynamic light scattering (DLS). Figure S2: The normalized and time-dependent optical absorption *α*(t)/*α*(0) of the characteristic peaks of MO and MB after mixed with S500. Figure S3: The time-dependent UV-Vis spectra of (a) methyl orange and (b) methylene blue after being mixed with S500 Cu*_x_*Fe*_y_*O*_z_* NPs in the dark for 24 h. Figure S4: Concentration-dependent antimicrobial activity of S200 and S500 samples against *E. coli* B. Figure S5: XRD patterns of the control (a) Fe_2_O_3_ and (b) Cu_2_O NPs.
