# Supplementary material for: Highly Efficient Antimicrobial Activity of CuxFeyOz Nanoparticles against Important Human Pathogens
Source: Nanomaterials (Basel). 2020 Nov 20;10(11):2294. doi: 10.3390/nano10112294 (PMC7699552; doi:10.3390/nano10112294)
Supplement: Supplementary file 1 [file nanomaterials-10-02294-s001.pdf]

nanomaterials

MDPI

Supporting Information

Highly Efficient Antimicrobial Activity of $Cu_xFe_yO_z$ Nanoparticles Against Important Human Pathogens

Lu Zhu [1], David W. Pearson [2], Stéphane L. Benoit [3], Jing Xie [4], Jitendra Pant [1], Yanjun Yang [1], Arnab Mondal [1], Hitesh Handa [1], Jane Y. Howe [5], Yen-Con Hung [4], Jorge E. Vidal [6], Robert J. Maier [3], and Yiping Zhao [2,*]

1 School of Chemical, Materials and Biomedical Engineering, College of Engineering, University of Georgia, Athens, GA 30602, USA; zhulu5466@gmail.com (L.Z.); jp50261@uga.edu (J.P.); YanjunYang@uga.edu (Y.Y.); Arnab.Mondal@uga.edu (A.M.); hhanda@uga.edu (H.H.)
2 Department of Physics and Astronomy, University of Georgia, Athens, GA 30602, USA; davidwpuga@gmail.com
3 Department of Microbiology, University of Georgia, Athens, GA 30602, USA; stefbens@uga.edu (S.L.B.); rmaier@uga.edu (R.J.M.)
4 Department of Food Science & Technology, University of Georgia, Griffin, GA 30223, USA; jing.xie25@uga.edu (J.X.); yhung@uga.edu (Y.-C.H.)
5 Department of Materials Science & Engineering, University of Toronto, Toronto, ON M5S 1A1, Canada; jane.howe@utoronto.ca
6 Department of Microbiology and Immunology, University of Mississippi, Jackson, MS 39216, USA; jvidal@umc.edu
* Correspondence: zhaoy@uga.edu

S1. Summary of different bacteria strains used in this work

Table S1. Bacterial strains used in this study

Strain	Relevant characteristics	Source or Reference
	Gram-negative	
Escherichia coli		
strain B	Nonpathogenic	ATCC (11303)
strain O157:H7 E009	Nalidixic acid resistant	Ref. [1]
Helicobacter pylori		
strain X47	Mouse-colonizing strain	Ref. [2]
Klebsiella pneumoniae		
strain 4/484	Nosocomial, Multidrug-Resistant	Ref. [3]
strain 1100975	Multidrug resistant (New Delhi metallo-beta-lactamase 1)	ATCC (BAA2472)
Salmonella enterica Typhimurium		
strain 700408	Multidrug resistant	ATCC (700408)
Shigella flexneri		
strain 2457T	Serotype 2a	ATCC (700930)
	Gram-positive	
Listeria monocytogenes		
strain Scott A	Human isolated from an outbreak linked to milk	Ref. [4]
Staphylococcus aureus		
Strain Rosenbach	Pathogenic	ATCC (6538)

ATCC: American Type Culture Collectio

S2. Size characterization of different $Cu_xFe_yO_z$ NP samples

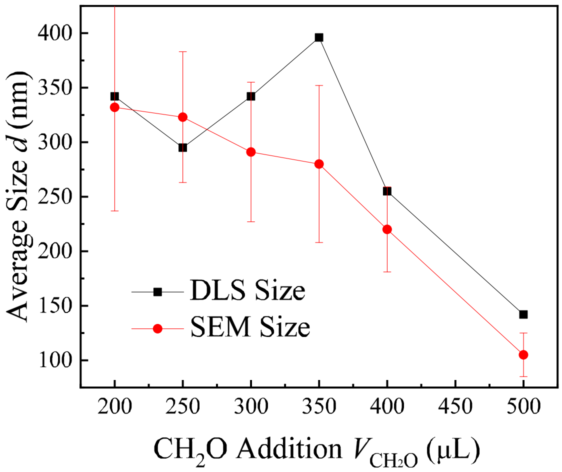

Figure S1. Size comparison between SEM data and dynamic light scattering (DLS). Different $Cu_xFe_yO_z$ NP samples are designated by formaldehyde (μL) addition during synthesis.

Table S2 Summary of the properties of $Cu_xFe_yO_z$ NP samples

Sample of $Cu_xFe_yO_z$ as (L) of Formaldehyde	XRD Crystallite Size (nm)	SEM Average Size (nm)	Zeta Potential (mV)	10 hour MO Concentration <u>*Reduction*</u> *(Dark)*	24 hour MO Concentration <u>*Reduction*</u> *(Dark)*
200	35.3	300	20.9	3%	22%
250	35.3	270	-13.3	38%	71%
300	42.4	220	-8.7	35%	76%
350	21.2	220	13.8	35%	85%
400	23.6	230	14	28%	61%
500	53	130	-10.5	83%	90%

S3. Catalytic activity of S500 against MO and MG

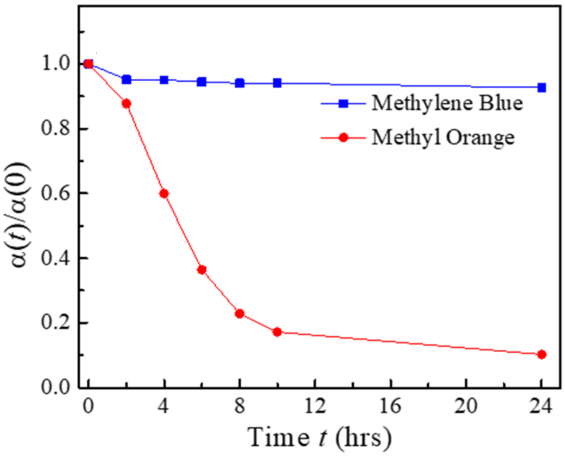

Figure S2. The normalized and time dependent optical absorption $\alpha(t)/\alpha(0)$ of the characteristic peaks of MO and MB after mixed with S500. The experiments were performed under dark.

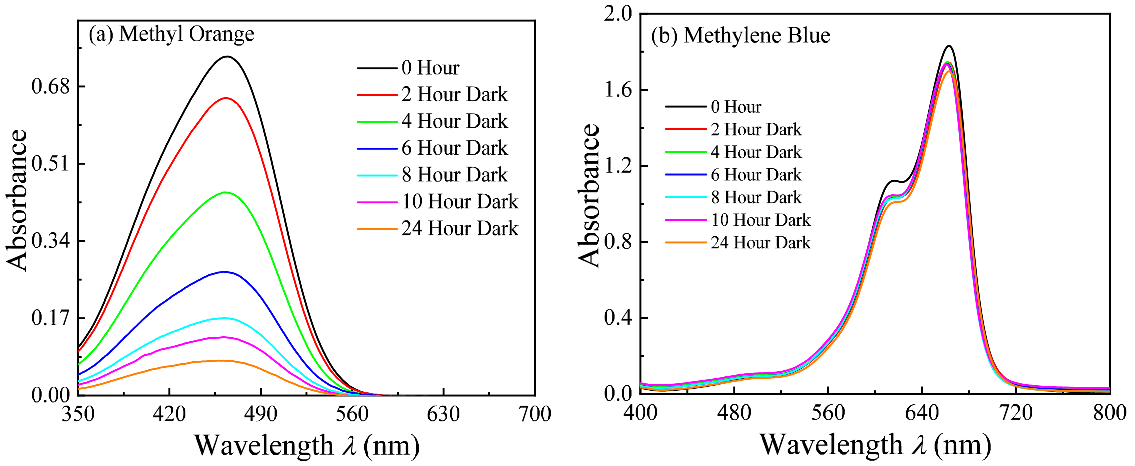

Figure S3. The time dependent UV-Vis spectra of (a) methyl orange and (b) methylene blue after mixed with S500 $Cu_xFe_yO_z$ NPs in dark for 24 hours.

S4. Antimicrobial tests for different concentrations of S200 and S500

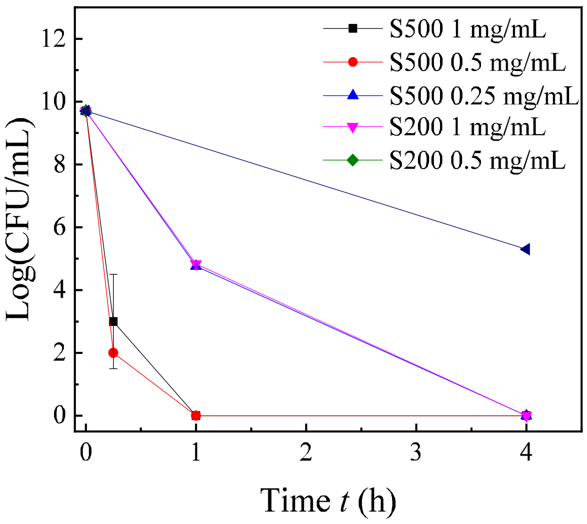

Figure S4. Concentration-dependent antimicrobial activity of S200 and S500 samples against *E. coli* B.

S5. Control samples and their properties

The control Fe_2O_3 and Cu_2O NPs were synthesized under the same microwave assisted hydrothermal condition with 500 μL 37% formaldehyde (Unfortunately we could not obtain $CuFeO_2$ NPs under similar conditions or other conditions). We kept the ion molar amount the same for the synthesis, i.e., for Fe_2O_3 NPs, 1 mmol $Fe(NO_3)_3 \bullet 9H_2O$ (0.404 g) was used; while for Cu_2O NPs, 1 mmol $Cu(NO_3)_2 \bullet 3H_2O$ (0.242 g) was used. The average sizes of the Fe_2O_3 and Cu_2O NPs were determined to be 632 and 832 nm by a dynamic light scattering measurements. The composition of the resulted NPs was determined by XRD, as shown in Fig. S5. The control Fe_2O_3 NPs are confirmed as pure Fe_2O_3 while the Cu_2O NPs contain small amount of CuO.

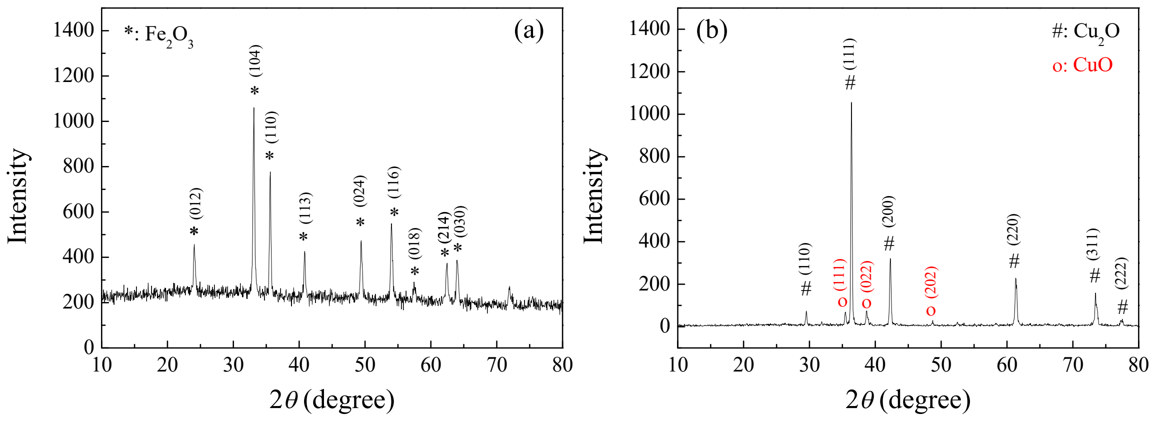

Figure S5. XRD patterns of the control (a) Fe_2O_3 and (b) Cu_2O NPs.

References

1. Yemmireddy, V.K.; Hung, Y.-C. Selection of photocatalytic bactericidal titanium dioxide (TiO2) nanoparticles for food safety applications. *LWT - Food Science and Technology* **2015**, *61*, 1-6, doi:10.1016/j.lwt.2014.11.043.
2. Veyrier, F.J.; Ecobichon, C.; Boneca, I.G. Draft Genome Sequence of Strain X47-2AL, a Feline Helicobacter pylori Isolate. *Genome announcements* **2013**, *1*, doi:10.1128/genomeA.01095-13.
3. Ostria-Hernandez, M.L.; Juárez-de la Rosa, K.C.; Arzate-Barbosa, P.; Lara-Hernández, A.; Sakai, F.; Ibarra, J.A.; Castro-Escarpulli, G.; Vidal, J.E. Nosocomial, Multidrug-Resistant Klebsiella pneumoniae Strains Isolated from Mexico City Produce Robust Biofilms on Abiotic Surfaces but Not on Human Lung Cells. *Microbial Drug Resistance* **2018**, *24*, 422-433.
4. Kim, C.; Hung, Y.-C.; Brackett, R.E. Efficacy of electrolyzed oxidizing (EO) and chemically modified water on different types of foodborne pathogens. *International Journal of Food Microbiology* **2000**, *61*, 199-207.

© 2020 by the authors. Licensee MDPI, Basel, Switzerland. This article is an open access article distributed under the terms and conditions of the Creative Commons Attribution (CC BY) license (http://creativecommons.org/licenses/by/4.0/).
